# The Importance of Nonlinear Transformations Use in Medical Data Analysis

**DOI:** 10.2196/medinform.7992

**Published:** 2018-05-11

**Authors:** Netta Shachar, Alexis Mitelpunkt, Tal Kozlovski, Tal Galili, Tzviel Frostig, Barak Brill, Mira Marcus-Kalish, Yoav Benjamini

**Affiliations:** ^1^ Department of Statistics and and Operations Research Tel Aviv University Tel Aviv Israel; ^2^ Pediatric Neurology Dana-Dwek Children's Hospital Tel Aviv Sourasky Medical Center Tel Aviv Israel; ^3^ School of Medicine Sackler Faculty of Medicine Tel Aviv University Tel Aviv Israel; ^4^ Sagol School of Neuroscience Tel Aviv University Tel Aviv Israel

**Keywords:** data mining, statistics, preprocessing, medical informatics, health informatics, big data, transformations

## Abstract

**Background:**

The accumulation of data and its accessibility through easier-to-use platforms will allow data scientists and practitioners who are less sophisticated data analysts to get answers by using big data for many purposes in multiple ways. Data scientists working with medical data are aware of the importance of preprocessing, yet in many cases, the potential benefits of using nonlinear transformations is overlooked.

**Objective:**

Our aim is to present a semi-automated approach of symmetry-aiming transformations tailored for medical data analysis and its advantages.

**Methods:**

We describe 10 commonly encountered data types used in the medical field and the relevant transformations for each data type. Data from the Alzheimer’s Disease Neuroimaging Initiative study, Parkinson’s disease hospital cohort, and disease-simulating data were used to demonstrate the approach and its benefits.

**Results:**

Symmetry-targeted monotone transformations were applied, and the advantages gained in variance, stability, linearity, and clustering are demonstrated. An open source application implementing the described methods was developed. Both linearity of relationships and increase of stability of variability improved after applying proper nonlinear transformation. Clustering simulated nonsymmetric data gave low agreement to the generating clusters (Rand value=0.681), while capturing the original structure after applying nonlinear transformation to symmetry (Rand value=0.986).

**Conclusions:**

This work presents the use of nonlinear transformations for medical data and the importance of their semi-automated choice. Using the described approach, the data analyst increases the ability to create simpler, more robust and translational models, thereby facilitating the interpretation and implementation of the analysis by medical practitioners. Applying nonlinear transformations as part of the preprocessing is essential to the quality and interpretability of results.

## Introduction

### Medical Data Analysis

The volume of data collected these days is constantly growing and is expected to reach 44 zettabytes by 2020 [1], and medical data are rapidly catching on to this trend. Informed use of data collected from the entire population, in a way that can lead to providing better treatment to each patient, is a challenging goal. Unlike the traditional way of collecting data for a specific purpose, big data and its relevant subsets are analyzed for multiple purposes, in multiple ways by means of statistical models, data mining algorithms, machine learning methods, and others. Moreover, the accessibility of big data through easier-to-use platforms, such as the open source KNIME Analytics Platform or older commercial software such as SPSS Modeler, will allow practitioners who are not expert data analysts to get answers by analyzing big data. Systems designed to specifically cater to a wider range of less-sophisticated data analysts in the field of health informatics are becoming more common. For example, the Medical Informatics Platform of the European Human Brain Project is an innovative data analysis system that “provides an interface through which clinicians, neuroscientists, epidemiologists, researchers but also health managers and even the general public can access and analyze imaging and clinical data currently locked in hospital and research archives and public databases.” We demonstrate that transformation-based preprocessing is an enhancer tool important for even simple questions using simple tools. Incorporating transformations into the preprocessing stage is essential in order to enable less-sophisticated data analysts to obtain informative and relevant results by simple means.

With the ever-increasing numbers of variables and subjects, there is a notion in the general public that simply the amount of data will reveal all there is to understand from it. This is not necessarily so. Data analysis can be greatly simplified when (1) the distribution of a variable (feature) is symmetric across subjects, (2) variability is stable across different conditions, (3) relationships are linear between variables of interest, and (4) models are additive rather than having a more complex structure. The need for these desirable properties can be circumvented by very complex modeling, which is feasible in data-rich research, as discussed below. However, such complex modeling leads to results that are harder to interpret and less appropriate for generalizing, and the ability to extrapolate beyond the given data or to set thresholds with diagnostic values is reduced. In addition, complex modeling requires a high level of expertise in data analysis—a level most physicians and health specialists cannot devote enough time to achieve, even if they are interested in acquiring quantitative answers to their questions.

In practice, data as extracted and measured are not usually characterized by the above four desirable properties. Moreover, the widely used essentially linear transformations such as normalization or Z score are practically linear transformations and therefore cannot change the symmetry or linearity. It is well known that income information is better analyzed after applying a logarithmic transformation, and the same is true for concentrations of substances in body fluids or the use of log odds for risk modeling. It is similarly recognized that gene expression data should be log-transformed and its variability stabilized before it can be compared across subjects and conditions. This remains true no matter what role it plays in the modeling effort: whether it is the variable of interest to be analyzed or used for prediction and explanation of another quantity of interest. The principles that the data need not remain in their original scale of measurement and that monotone transformations of variables can be used to facilitate the interpretation and generalization are well accepted in specific research situations. It is a cornerstone of the Exploratory Data Analysis approach to analyzing small and medium datasets [2].

The above principle is undervalued and rarely used when addressing bigger and more complex datasets. A search in the Web of Science database for the terms “classification OR prediction OR clustering” published in one of the three leading medical informatics journals (*Journal of the American Medical Informatics Association*, *International Journal of Medical Informatics*, *BMC Medical Informatics and Decision Making*) between January 2013 and April 2015 yielded 226 articles. This list was further screened manually, and 43 articles that specifically dealt with clinical data analysis were selected. Of these, 16 used data mining and machine-learning algorithms for classification of clinical data. Only one article mentioned performing a preprocessing stage, which consisted only of correcting misalignments, missing data, and the selection of the most predictive variables (see Multimedia Appendix 1). This does not necessarily imply that all should have, but rather reflects the common practice.

### The Importance of Symmetry-Improving Nonlinear Transformations in Preprocessing

We demonstrate that transforming a variable so that its distribution across individuals is approximately symmetric goes a long way towards achieving the other goals for which transformations are useful: stabilizing the variance across conditions, assuring linearity in the relationship between variables, and the additivity of the response of interest [2,3]. The symmetric distribution need not be Gaussian, but symmetry does imply that summaries of such transformed distribution will be close to Gaussian, allowing the call of outliers and the setting of confidence statements of regression coefficients and other inferences to be more justified. Moreover, symmetry is highly desirable when computing distances on many features reflecting different underlying entities, where skewed distributions may hide important differences that are not in the tails.

Sometimes it is not feasible to transform to symmetry because the variable has a more complex structure, such as multimodality (“bumps” or “humps”). Even then, transforming in a way that causes the main body of data to be in a symmetric hump is advantageous.

Limiting the search for appropriate transformation at the preprocessing stage towards symmetry only becomes essential when confronted with big data. The detailed inspection of the relationship between every pair of variables, inspecting the residuals from every model under consideration, for lack of linearity or homogeneity of variability is impossible. Nor is it known ahead of time what the problem of interest to a future user will be. In contrast, when searching for symmetry-inducing transformations at the preprocessing stage, the number of manual inspections is at most linear in the number of variables and thus becomes feasible.

We therefore propose a semi-automated practice for symmetry-targeting preprocessing that enables the data scientist to select an appropriate transformation for each variable in the dataset, thereby allowing the analysis of thousands of variables in an efficient and reproducible manner.

It is important to acknowledge that for every specific analysis, more complex procedures may be used to overcome the limitations discussed above, including nonlinear methods based on splines or loess, robust methods that ignore outliers, and hierarchical methods that capture interactions [4-6]. These and other methods are comprehensively described in Hastie et al [7]. Even such high-powered users will enjoy a better starting point post transformation. The more casual ones will be saved from disaster.

## Methods

### Monotone Transformations

The monotone transformations we use belong to the power transformations family, where *x* is transformed to *x*^p^. We recommend Tukey’s ladder of re-expression, where squares, square root, their reciprocals, and their likes are used [2]. Formally, use *x*^p^, where *p=­m/n, n=1,2,3* and *m=–2,–1,0,1,2*. In all formulations, *p*=0 means using the logarithmic transformation (the base is not important, so base 2, the natural base, and base 10 are usually used). We prefer this ladder, where a discrete set of powers is used, over allowing any *p*, as in the Box-Cox formulation [8]. In the latter, *p* is tailored to optimize a specific goal, while at the preprocessing stage, no single goal is clear at the outset. Tukey’s ladder includes all transformations that are known to be particularly useful such as log, square root and the cubic roots, and their inverses. Note that when *p* is negative, the transformation monotonically decreases rather than increases. In many cases, the meaning remains intuitive. For example, when the time to pass 1 meter is measured, once transformed by (time)^–1^ it is merely the speed that is recorded. The longer the time, the lower the speed.

The above transformations are further combined with other transformations that are tailored to the type of variable encountered. Tukey’s taxonomy of measured variables [2] as is applicable to medical variables is presented, as well as the adequate transformation to be used for each type in conjunction with the power family. This taxonomy is helpful when dealing with a dataset that encompasses a large and varied number of variables. Further explanations, the mathematical and statistical reasoning, and the transformation equations can be found in Multimedia Appendix 2.

### Tukey’s Taxonomy Applied to Medical Variables and Their Appropriate Transformations

Amounts are measurements taking any positive value *y*≥0 (eg, serum protein level). Some measurements are inherently bounded from below, that is, *y*≥C, in which case *y*‒C is a valid amount. In order to arrive at a symmetric distribution for amounts, we can use any power transformation directly (see Multimedia Appendix 2 for handling amounts that include zeros).

Counts are counts of units and therefore can take only integer values 0,1,2,… (eg, number of adverse events, days under antibiotic treatment). Counts tend to be right-skewed and have variance increasing with their mean, stemming from the Poisson nature of their distribution. The square root is a variance stabilizing transformation for Poisson counts and often achieves symmetry.

Ratio is one amount *x* divided by another amount *y*, that is, r=*x*/*y* (eg, protein/creatinine ratio in the urine). Ratios often require log transformations. This then takes the form of log(r)=log(x)–log(y). Other power transformations can replace log, in the form of *x*^p^‒*y*^p^, if the two elements are available.

Fraction is a ratio of one amount *x* to some other amount *y*, which is always larger, that is, 0≤r=*x*/*y*≤1 (eg, fraction of a blood vessel that is clotted). Usually deciding on the property measured by the ratio *r* is equivalent to deciding to measure its complement 1‒r (instead of the fraction of a blood vessel that is clotted, one could define the fraction that is open). Log (r/1‒r), also known as the “logit” transformation, is the most useful (the widely used log odds for comparing morbidity).

Counted fraction is the fraction of counts, counting how many out of how many have some property (n out of m). It is therefore bound between 0 and 1 (eg, the number of patients admitted on a day due to a specific symptom out of the total number of admitted patients that day). Here too, the most useful transformation is the logit transformation: log (n+⅓/m–n+⅓). We add a constant c=⅓ to avoid dividing by zero [2].

Amounts that are inherently bounded a≤y≤b, are essentially fractions, given by 0≤r=(y‒a)/(b‒a)≤1 (eg, the length of time a child sleeps per day). We first write them as fractions and then transform them as discussed above.

Counts that are inherently bounded l≤n≤m, are essentially counted fractions, given by 0≤r=n‒l)/(m‒l)≤1 (eg, the number of correctly answered questions in a questionnaire), and as such, they should be handled in the same manner explained for counted fractions, log(n‒l+⅓)/(m‒n+⅓).

Difference in amounts, counts, or fractions may take positive or negative values (eg, the difference between the number of words forgotten in an immediate recall assignment and number of words forgotten in a delayed recall assignment). Differences are best handled by transforming the subtracted variables separately, be they amounts or counts. If the two variables that are differenced are not available, the difference variable should rarely be transformed. Instead, it can practically be only positive where it should be handled as amount.

Ranks are bounded counts of how many are below and equal the observation out of the total number ranked (eg, the rank of a specific symptom-describing word out of all descriptive words used). They will be treated exactly as bounded counts.

Ordinal variables are variables whose values are named categories that can be naturally ordered (eg, everyday cognition assessment taking the values 1-4). Each category can be assigned a numeric value (similar to ranks with ties) depending on the proportion of cases in the category and below it, compared to the background of reference distribution. An explicit formula for a logistic reference distribution is given in Multimedia Appendix 2.

The medical information dataset should, therefore, be accompanied by a metafile, specifying for each variable its type (according to the above 10-item list), its context-related lower and upper bounds (when these exist). Finally, an indicator of whether the variable should be reversed, in order to ease interpretation should be added, so that variables carrying similar meaning, say “healthy,” are presented in the same direction.

### A Semi-Automated Approach to Choosing Symmetry-Targeted Transformations

With the above information at hand, we recommend that the choice of transformations be performed in a semi-automated manner. The automated part can be guided by the measure of skewness that directed the analyst towards a few alternative transformations around the power *p* that was calculated. Yule’s measure of skewness [9] (also known as Bowley’s) usually serves this purpose well, with a value of 0 for symmetric data:

sk=0.5(*m*_3_+*m*_1_)– 
*m*_2_/0.5*(*m*_3_–*m*_1_)

where *m*_1_, *m*_2_, and *m*_3_ are the lower quartile (ie, the 25^th^percent quantile), the median, and the upper quartile (ie, the 75^th^percent quantile), respectively. The interquartile range 0.5*(*m*_3_–*m*_1_) that is a measure of the spread is equal to the median absolute deviation for exactly symmetrical distributions.

A less-resistant version of the Yule index can serve well, even when the data consist of bounded counts over a small range (see Multimedia Appendix 2).

The automatic search is combined with subjective assessment in the following way. For a given variable *x*_i_, if |sk_i_|˂*c*, no transformation was needed, and the next variable is inspected. As a rule of thumb, a recommended setting is *c*=0.1 when the number of cases is bigger than 180, and larger when the number is smaller: 0.12 for 120 and 0.15 for 80. For all skewed variables, according to the type of variable as listed in the metafile, histograms are computed and displayed for the original values and the power transformation over Tukey’s ladder that makes the variable most symmetric, as well as of closely related transformations. Unlike the Box-Cox transformations that are flexibly chosen, those on Tukey’s ladder are all easily interpretable and more robust. Moreover, we use the chosen transformation as the starting point: the plots of the distribution following these potentially useful transformations are shown with the corresponding skewness measure. Finally, the researcher should choose which of the transformations (if any) best suits the specific need. The type of transformation performed on each of the variables is registered in order to enhance reproducibility.

### Data

The above approach was implemented in R and used to preprocess data obtained from the Alzheimer’s Disease Neuroimaging Initiative (ADNI). Alzheimer’s disease (AD) is the most common form of dementia. There is currently no known treatment nor one that slows the progression of this disorder [10]. Therefore, finding pathways for treatment is a major research effort. ADNI was conceived at the beginning of the millennium as a North American multicenter collaborative effort funded by public and private bodies, in order to facilitate a progression in the understanding, assessment, and treatment of AD. The initiative obtains data on patients of normal cognitive state, early and late mild cognitive impairment, significant memory concern, and AD. Clinical, neuropsychological, biological markers, imaging, and genetic data are collected on the patients [10]. The clinical measurements were obtained from multiple data tables, extracted from the ADNIMERGE R package (version 0.0.1 downloaded June 2014). A no-missing-values-subset of the ADNI data with 185 potentially explanatory variables and a target variable—the assigned diagnosis on 658 participants—was used for the demonstration.

A second dataset involved 1575 Parkinson’s disease (PD) patients and their first degree relatives assembled in the Tel Aviv Medical Center: 1185 idiopathic PD patients, 164 PD patients who are carriers of the G2019S mutation in the *LRRK2* gene, and 226 PD patients who are carriers of mutations in the *GBA* gene. Patients were followed between 2008 and 2015. Each subject underwent a battery of medical exams and questionnaires ranging from demographic to physical, cognitive, and performance-based assessments. These included physical and neurological examinations as well as clinical assessment questionnaires, for example, the Unified Parkinson’s Disease Rating Scale (UPDRS). Other symptoms were evaluated by the Beck Depression Inventory, Geriatric Depression Scale, Scopa-Aut, and more. Overall, 772 different measures were made available to us.

In addition to the clinical datasets described above, the benefits of transformations for cluster analysis were demonstrated using simulated, rather than real research data. First, most clustering algorithms require the unknown number of clusters as input; this number is known in the simulated data scenario. Second, assessing the accuracy of the clusters is not straightforward, as the real separation into subgroups is absent. When the data are generated according to a known clustered structure, the accuracy of the results can be easily measured using, for example, using the adjusted Rand index [11].

We constructed a simulated dataset of the clinical situation described as originally three assigned diseases: A, B, and C. The “real” situation is that disease A has two subtypes A1, A2; Diseases B and C are actually two subtypes of disease BC. The data contained 100 continuous, discrete, and binary features, of which 7 features define 6 disease subgroups (A1, A2, B, C, BC, and Normal). Each feature was created symmetrically and then transformed in order to exhibit a skewed distribution. The clustering analysis was performed on both the original and the transformed data.

In order to facilitate the use of the study methodology, an implementation in an R shiny application was developed [12]. The software allows graphic selection of variable transformations. The application workflow is as follows: (1) the user uploads a dataset and metadata file that defines the variables’ properties, (2) the software output is a set of the most suitable transformations for each variable, presented in the form of a histogram/bar plot (with a density curve when appropriate), (3) the user selects the desired transformation and can download the transformed dataset, and (4) variables are displayed in a descending order according to their Yule index and relevance.

The application allows data transformation regardless of modeling procedure. Visualization of the choices and selection made allows the user to transform hundreds of variables in a matter of minutes, without the burden of using scripts, remembering the available transformations, and tuning parameters.

## Results

### Findings

The improvement in the results of statistical analysis methods such as analysis of variance and linear regression, after the preprocessing methods were applied, are described below. Additional benefits gained by symmetry-targeting transformations, such as reduction of variance variability and linearity, are presented next. A dissimilarity matrix is usually based on some distance metric and may therefore be highly deformed due to the absence of symmetry. In a highly skewed variable, the distances between the elements in the bulk of the data will be substantially smaller than the distances between the very few large ones, the latter marring the importance of differences for most of the observations. The improvement in clustering is demonstrated using simulated data in the concluding section of the Results.

### Transforming Medical Data for Symmetry

**Figure 1:**
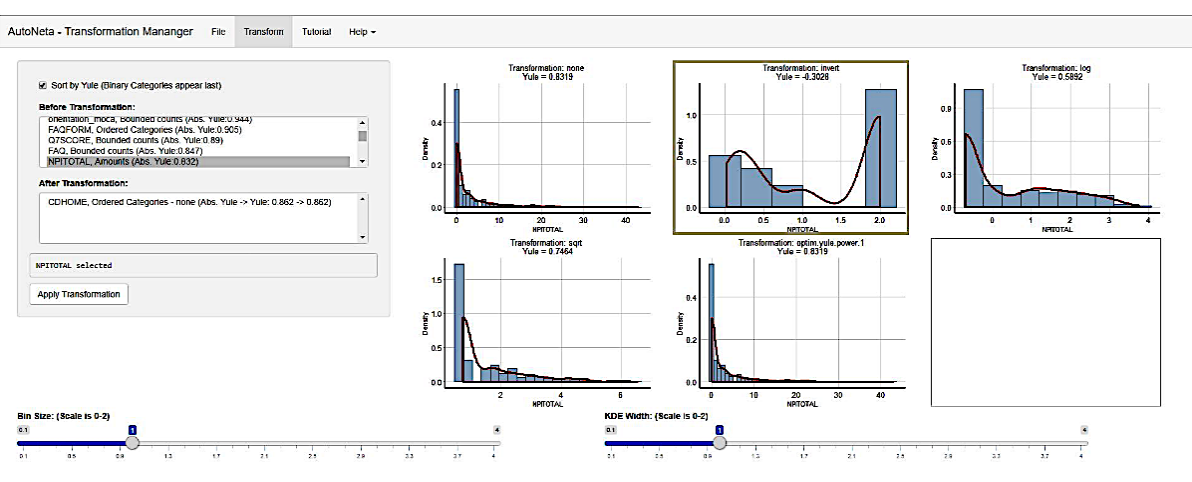
Screenshot of semi-automated transformation workflow application of Alzheimer’s Disease Neuroimaging Initiative (ADNI) patients' data variable neuropsychiatric inventory total score distribution.

The first example is the semi-automated output for the variable NPITOTAL (neuropsychiatric inventory total score) in the ADNI cohort represents the total score of the psychiatric inventory exam. Examinations are shown in Figure 1. Since the original distribution was right skewed, the application offers performing an inverse transformation, a log transformation, and a power of 0.5 transformation (namely square root transformation). In this case, it appears that the inverse transformation is the most suitable.

**Figure 2:**
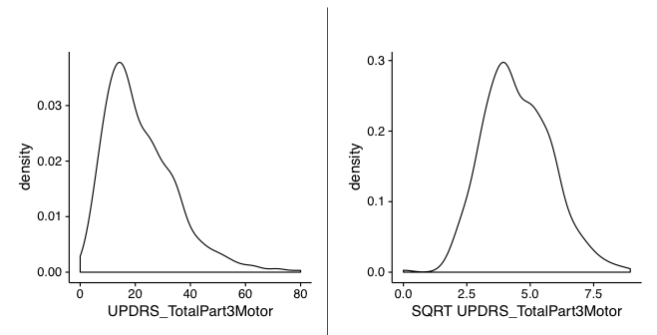
Unified Parkinson's Disease Rating Scale (UPDRS) Part 3 (motor part): variable raw data distribution, variable after sqrt transformation.

The variable UPDRS Part 3 measures the score for the integrated motor-related condition of PD patients. Figure 2 displays the right-skewed frequency histogram (density plot) for the raw values, and the appropiate square root transformation.

Even after transformation, the distributions of the variables are not necessarily perfectly symmetrical, but their skewness decreased substantially.

### The Gains From Striving for Symmetry

Although the selected transformations relied only on symmetry as the desirable target, they demonstrate how helpful these can be in achieving other goals as well.

**Figure 3:**
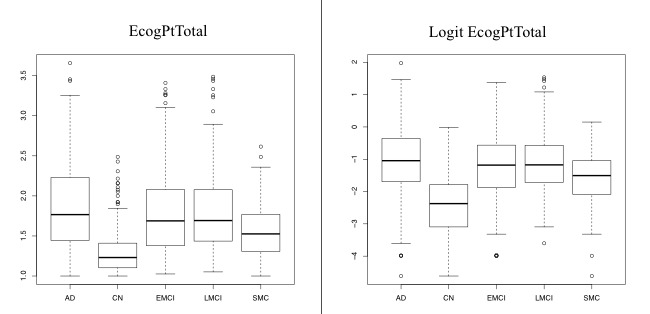
EcogPtTotal (everyday cognition participant total score) variable raw data over Alzheimer’s Disease Neuroimaging Initiative (ADNI)'s five diagnosis categories before and after Logit transformation.

Analysis of variance is most frequently used in order to check for differences in the means between three or more groups. It is therefore a useful tool when screening for variables, which have some marginal association with categorical variables such as the diagnosis groups. Figure 3 shows the variable of the total score of Everyday Cognition as self-stated by the participant compared to 10 years before (EcogPtTotal [everyday cognition participant total score]) before and after the above-mentioned symmetry transformation. The raw values over the five diagnosis categories assigned by the ADNI research using boxplots are compared in Figure 3. The variability (as reflected by the box lengths and the ranges) changes between the groups: the higher the median (reflecting the center), the higher the variability. The same groups are displayed for the transformed variable on the right side of Figure 3. Not only is the distribution within each group symmetric, but the dependency of variability on the median disappears and is quite homogeneous across the diagnosis groups.

**Figure 4:**
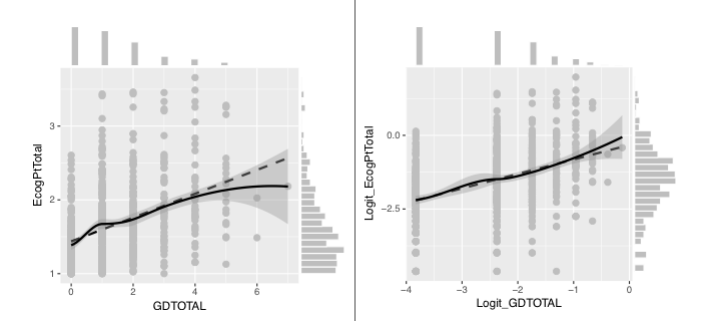
Relationship between EcogPtTotal (everyday cognition participant total score) and GDTotal (score for the integrated geriatric depression scale) raw values and after transformations.

The linearity of relationships is a desirable property, allowing simpler models and better predictions outside the range of available data. These can later be extrapolated and easily explained. The relationship between EcogPtTotal and geriatric depression total score (GDTotal; score for the integrated geriatric depression scale in the ADNI data) is explored and shown in Figure 4 for the raw variables. The relationship is shown after the variables were transformed, using symmetry of each variable separately as our only criterion. On the raw scales, the monotone relationship (solid line) is far from linear (dashed line) and is rather concave. On the transformed scales, the relationship is quite linear.

Increase of the stability of variability following the transformation is beneficial as well. The distance between the two quartile lines (grayed area) increases with the progression in GDTotal value (Figure 4). In contrast, after transformation, the distance is stable all across the relationships (Figure 4). It should be emphasized that even though the transformation was chosen to approximately symmetrize the original variable, it is the distribution of the residuals that gained symmetry and homogeneous variability—the property we need. This fortunate outcome is likely to happen across many modeling efforts.

Both linearity of relationships and increase of stability of variability can be used to demonstrate the practical importance of simplicity of a relationship. Suppose we want to identify unusually low self-assessed deterioration, not explained merely by depression, which may indicate a more serious cognitive deterioration. The physician should insert the result of GDTotal into a computer program (that expresses the nonlinear relationship), get the typical EcogPtTotal (standard deviation 2), calculated for that specific GDTotal value, and check whether the patient’s value is above it. Instead, if the measurement is reported in the transformed way, the physician can immediately see whether the patient is above this threshold and by how much. The stability of the calculation and the possibility to extend the relationship into the region where only a few measurements exist are substantially improved.

### The Gains in Cluster Analysis by Aiming for Symmetry

Recall that a simulated dataset of the clinical situation described above was generated. First, we performed a 2-step analysis of the original symmetric data: (1) select a subset of the clinical measurements using stepwise selection with false discovery rate control for q=0.05 [13], and (2) perform cluster analysis using the partition around medoids (K-medoids) algorithm [14] with Manhattan distance matrix. Then, we “damaged” some of the meaningful features by monotone transformation so that their distribution became asymmetric and repeated the analysis (see Multimedia Appendix 2 for further details on the simulation). The lack of symmetry following the process is clearly observed and is further emphasized by the value of the Yule index reported below the histograms. We evaluated the quality of the clustering using the confusion matrices and the adjusted Rand index. The results were conclusive: while symmetric variable distribution has little to no effect on the selection results, it is crucial for the clustering step. The confusion matrices and the adjusted Rand values that demonstrate the results are presented in Table 1. The use of different subset selection methods, different clustering algorithms, or different distance matrices had no effect on the direction of the results and had only a minor, if any, effect on its magnitude.

**Table 1:** Comparison of cluster analysis results^a^.

True cluster	Assigned membership using symmetric data^b^						Assigned membership using skewed data^c^					
	1	2	3	4	5	6	1	3	2	4	6	5
0	200	0	0	0	0	0	194	0	6	0	0	0
1.1	0	200	0	0	0	0	0	200	0	0	0	0
1.2	0	0	200	0	0	0	0	63	137	0	0	0
2	0	0	0	200	0	0	0	0	0	140	1	59
3	0	0	0	0	199	1	2	0	0	2	196	0
23	0	0	0	0	6	194	0	1	0	91	7	101

^a^Confusion matrix of real group membership versus assigned cluster using K-medoids algorithm and Manhattan distance matrix used on symmetric (left) and asymmetric (right) features.

^b^Adjusted Rand=0.986.

^c^Adjusted Rand=0.681.

## Discussion

### Principal Results

The importance of data transformation in the preprocessing stage for any big data analysis, especially in medicine, is presented in this paper. We focused on the use of data transformation as the lesser known part of data preparation. Reviewing articles in medical informatics, we noticed that the preprocessing methods are not always mentioned. Even when they are performed, in many cases the process does not include variable transformations (Multimedia Appendix 1). It is our strong belief that the preprocessing steps taken have to be detailed as this may be crucial to both achievement of valuable results and to their reproducibility.

As seen in the examples from the large and robust databases of clinical and genetics information of AD and PD patients, the use of transformations results in simpler, linear, additive models with homogeneous variability. This enhances the ability to extrapolate and produce more accurate predictions, with better distance calculations and reduced complexity facilitating the integration into systems and devices. As shown, performing data analysis without transformations is possible but may require complex nonlinear models to explain the data.

The purpose of data analysis is to discover new insights. In this sense, the interpretation is an essential factor in the success of a data analysis process. The described methods increase the translational ability of the results for clinicians. They provide the possibility to apply simpler and more explainable models. The variables can be reverted to the original values before presenting and communicating the results. Methods that serve as a “black-box” have difficulties gaining the trust of physicians. This, in part, is due to the lack of transparent explanations of the process leading to the results.

Moreover, there are patterns in the data that only a human can notice. An example is a case where one transformation had the smallest skewness value, but the proper transformation was actually another (Figure 1). This situation occurred due to the “double hump” of the distribution of the data, where many subjects had a value of 0 while the rest were distributed symmetrically. This example is not rare in medical data and can easily be recognized by the human eye. Therefore, the methodology we propose aims to benefit from automation of most of the procedures but leaves the final decision to the human team of experts.

### Limitations

This paper is based on our experience in using the methodology and the application. As use of the application will grow, we expect to improve with the feedback of users. The use of the methodology requires understanding of the data and metadata characteristics—this might be time consuming but intimate familiarity with the data is key to successful data analysis.

The benefit of nonlinear transformations was demonstrated by simulation only for the purpose of clustering, as it is difficult to find a medical example of clusters of patients, well defined by a known set of variables, which can serve as a vehicle for such a demonstration.

### Conclusions

The use of nonlinear transformations as part of the preprocessing is important and affects the quality of the results. Symmetry-targeted transformations contribute significantly to other aspects of data analysis, enabling simpler and more translational models.

## Appendix

Multimedia Appendix 1 Articles of data mining and machine-learning algorithms used for classification of clinical data.

Multimedia Appendix 2 Transformations extended details.

## Abbreviations

AD: Alzheimer’s disease
ADNI: Alzheimer’s Disease Neuroimaging Initiative
EcogPtTotal: everyday cognition participant total score
GDTotal: geriatric depression total score
PD: Parkinson’s disease
UPDRS: Unified Parkinson’s Disease Rating Scale
