# Supplementary material for: The Importance of Nonlinear Transformations Use in Medical Data Analysis
Source: JMIR Med Inform. 2018 May 11;6(2):e27. doi: 10.2196/medinform.7992 (PMC5970282; doi:10.2196/medinform.7992)
Supplement: Multimedia Appendix 1 [file medinform_v6i2e27_app1.pdf]

Num	Journal	Title	Methods	Year	Reference
1	JOURNAL OF THE AMERICAN MEDICAL INFORMATICS ASSOCIATION	From vital signs to clinical outcomes for patients with sepsis: a machine learning basis for a clinical decision support system	NB , SVM, gaussian micture models, HMM	2014	Gultepe, Eren, et al. "From vital signs to clinical outcomes for patients with sepsis: a machine learning basis for a clinical decision support system."*Journal of the American Medical Informatics Association* 21.2 (2014): 315-325.
2	JOURNAL OF THE AMERICAN MEDICAL INFORMATICS ASSOCIATION	An interactive Bayesian model for prediction of lymph node ratio and survival in pancreatic cancer patients	bayesian	2014	Smith, Brian J., and James J. Mezhir. "An interactive Bayesian model for prediction of lymph node ratio and survival in pancreatic cancer patients."*Journal of the American Medical Informatics Association* 21.e2 (2014): e203-e211.
3	JOURNAL OF THE AMERICAN MEDICAL INFORMATICS ASSOCIATION	Machine learning-based prediction of drug-drug interactions by integrating drug phenotypic, therapeutic, chemical, and genomic properties	NB, decision trees, k-nearest, LR, SVM	2014	Cheng, Feixiong, and Zhongming Zhao. "Machine learning-based prediction of drug–drug interactions by integrating drug phenotypic, therapeutic, chemical, and genomic properties." *Journal of the American Medical Informatics Association* 21.e2 (2014): e278-e286.

4	JOURNAL OF THE AMERICAN MEDICAL INFORMATICS ASSOCIATION	A comparative analysis of methods for predicting clinical outcomes using high -dimensional genomic datasets	NB, MANB, FSNB, EBMC, LR, SVM, Lasso, ELM	2014	Jiang, Xia, et al. "A comparative analysis of methods for predicting clinical outcomes using high-dimensional genomic datasets." *Journal of the American Medical Informatics Association* 21.e2 (2014): e312-e319.
5	JOURNAL OF THE AMERICAN MEDICAL INFORMATICS ASSOCIATION	Medical decision support using machine learning for early detection of late-onset neonatal sepsis	NB, SVM, TAN, CART, RF, K-nearest, and 3 more.	2014	Mani, Subramani, et al. "Medical decision support using machine learning for early detection of late-onset neonatal sepsis." *Journal of the American Medical Informatics Association* 21.2 (2014): 326-336.
6	JOURNAL OF THE AMERICAN MEDICAL INFORMATICS ASSOCIATION	Predicting changes in hypertension control using electronic health records from a chronic disease management program	IG, RF	2014	Sun, Jimeng, et al. "Predicting changes in hypertension control using electronic health records from a chronic disease management program."*Journal of the American Medical Informatics Association* 21.2 (2014): 337-344.
7	JOURNAL OF THE AMERICAN MEDICAL INFORMATICS	Billing code algorithms to identify cases of peripheral artery disease from administrative data	LR	2013	Fan, Jin, et al. "Billing code algorithms to identify cases of peripheral artery disease from administrative data." *Journal of*

	ASSOCIATION				*the American Medical Informatics Association* 20.e2 (2013): e349-e354.
8	INTERNATIONAL JOURNAL OF MEDICAL INFORMATICS	Prediction of hospitalization due to heart diseases by supervised learning methods	SVM, adaboost, LR, NB, likelihood ratio	2015	Dai, Wuyang, et al. "Prediction of hospitalization due to heart diseases by supervised learning methods." *International journal of medical informatics*84.3 (2015): 189-197.
9	INTERNATIONAL JOURNAL OF MEDICAL INFORMATICS	Reducing unnecessary lab testing in the ICU with artificial intelligence	preprocessing, feature selection, classification - information gain	2013	Cismondi, Federico, et al. "Reducing unnecessary lab testing in the ICU with artificial intelligence." *International journal of medical informatics* 82.5 (2013): 345-358.
10	BMC MEDICAL INFORMATICS AND DECISION MAKING	A straightforward approach to designing a scoring system for predicting length-of-stay of cardiac surgery patients	NB	2014	Barbini, Paolo, et al. "A straightforward approach to designing a scoring system for predicting length-of-stay of cardiac surgery patients." *BMC medical informatics and decision making* 14.1 (2014): 1.

11	BMC MEDICAL INFORMATICS AND DECISION MAKING	Evaluating predictive modeling algorithms to assess patient eligibility for clinical trials from routine data	RF , others	2013	Köpcke, Felix, et al. "Evaluating predictive modeling algorithms to assess patient eligibility for clinical trials from routine data." *BMC medical informatics and decision making* 13.1 (2013): 134.
12	BMC MEDICAL INFORMATICS AND DECISION MAKING	Diabetic retinopathy risk prediction for fundus examination using sparse learning: a cross-sectional study	ridge, elastic net, lasso,	2013	Yoo, Tae Keun, and Eun-Cheol Park. "Diabetic retinopathy risk prediction for fundus examination using sparse learning: a cross-sectional study." *BMC medical informatics and decision making* 13.1 (2013): 106.
13	BMC MEDICAL INFORMATICS AND DECISION MAKING	Developing model-based algorithms to identify screening colonoscopies using administrative health databases	bayesian, multivariate LR, recursive paritioning	2013	Sewitch, Maida J., et al. "Developing model-based algorithms to identify screening colonoscopies using administrative health databases." *BMC medical informatics and decision making* 13.1 (2013): 1.
14	BMC MEDICAL INFORMATICS AND DECISION MAKING	Predicting out of intensive care unit cardiopulmonary arrest or death using electronic medical record data		2013	Alvarez, Carlos A., et al. "Predicting out of intensive care unit cardiopulmonary arrest or death using electronic medical record data." *BMC medical informatics and decision*

					making 13.1 (2013): 1.
15	BMC MEDICAL INFORMATICS AND DECISION MAKING	Evaluation of prediction models for the staging of prostate cancer	LR, RF, k-nearst	2013	Boyce, Susie, et al. "Evaluation of prediction models for the staging of prostate cancer." *BMC medical informatics and decision making* 13.1 (2013): 126.
16	BMC MEDICAL INFORMATICS AND DECISION MAKING	Bayesian predictors of very poor healt h related quality of life and mortality in patients with COPD	bayesian, 6 LR methods	2013	Ryynänen, Olli-Pekka, et al. "Bayesian predictors of very poor health related quality of life and mortality in patients with COPD." *BMC medical informatics and decision making* 13.1 (2013): 1.
