# Supplementary material for: The Importance of Nonlinear Transformations Use in Medical Data Analysis
Source: JMIR Med Inform. 2018 May 11;6(2):e27. doi: 10.2196/medinform.7992 (PMC5970282; doi:10.2196/medinform.7992)
Supplement: Multimedia Appendix 2 [file medinform_v6i2e27_app2.pdf]

1. The Tukey's family of transformations

Formally the power family of transformations is defined by f(x) = $(x^p-1)/p$ for any $p\neq 0$, and log(x) for p=0 since the limit as p->0 of f(x) is indeed log(x).

Since adding a constant and multiplying by a constant do not change the statistical properties, we resort to simply x^p.

For $p \leq 0$, e.g. log(x) and 1/x, the values of 0 pose a problem, which is addressed by adding a small constant *c* to all values. A good overall solution is to add the smallest number greater than 0 divided by 2: $\min\{x_i \mid x_i > 0\}/2$.

For counts the recommended choice is c=1/3

2. The transformation for ordered categorical variable

The categories are ordered, so for each category we can define:

$$q = \frac{¿ of\ obs. \in the\ category \vee below\ it}{Total\ observations};$$

$$p = \frac{¿ of\ obs.\ strictly\ below\ the\ category}{Total\ observations} \quad .$$

The transformed value for this category is:

$$q \log q + (1-q)\log(1-q) - [p \log p + (1-p)\log(1-p)]\ when\ 0<p<q<1;$$

$$q \log q + (1-q)\log(1-q)\ when\ p=0;$$

$$-[p \log p + (1-p)\log(1-p)]\ when\ q=1 \quad .$$

3. The approach of Emerson (1982) towards analyzing symmetry and the transformation to symmetry relies heavily on extreme quantiles. We therefor prefer relying on Yule's measure of skewness:

$$sk = \frac{0.5(m_3+m_1)-m_2}{0.5*(m_3-m_1)}$$

where m1, m2 and m3 are the lower quartile, the median and the upper quartile, respectively.

The measure is between -1 and 1, it indicates skewness to the right when positive, skewness to the left when negative, and 0 under symmetry.

We found a somewhat less resistant version of Yule’s index (Benjamini and Krieger, 1996) to serve well, when the data are bounded counts on a small range. The formula is the same as the above, but

$$m_1 = mean\left(x_{(1)} .. x_{\left(\frac{n}{4}\right)}\right); m_1 = mean\left(x_{\left(\frac{n}{4}+1\right)} .. x_{\left(n-\frac{n}{4}-1\right)}\right); m_3 = mean\left(x_{\left(n-\frac{n}{4}\right)} .. x_{(n)}\right)$$

where $x_{(1)} \leq x_{(2)} \leq x_{(n)}$ are the sorted data values (order statistics).
